# Supplementary material for: Regulation of antimycin biosynthesis by the orphan ECF RNA polymerase sigma factor σAntA
Source: PeerJ. 2014 Feb 6;2:e253. doi: 10.7717/peerj.253 (PMC3933326; doi:10.7717/peerj.253)
Supplement: Supplemental Information 1 — This document contains two supplementary tables and four supplementary figures as well as legends for each of these. [file peerj-02-253-s001.pdf]

FIGURE LEGENDS

Table S1. tblastn analysis suggests that σ^{AntA} is only encoded by antimycin-producing organisms taxa.

Table S2. Oligonucleotide primers used in this study.

Fig. S1. *antFG* and *antHIJKLMNO* comprise two divergently co-transcribed operons. (A) Schematic representation of the antimycin gene cluster. Narrow black arrows represent predicted (antAB, antCDE) operons and experimentally proven (antFG, antHIJKLMNO) operons and the direction of transcription. Horizontal bars denote the location of DNA targeted for RT-PCR analysis. (B) Agarose gel electrophoresis of RT-PCR products. RT+, cDNA reverse transcribed Δ*antA*/pIJ10257-*antA* RNA, RT- reverse transcriptase reaction mix with enzyme omitted. PCR products originating from cDNA template were cloned into pCRII-TOPO or pGEM-T Easy and sequenced.

Fig. S2. Clustal Ω alignment of σ^{AntA} orthologues identified a conserved Ala-Ala. Predicted σ_2 (green) and σ_4 domains are indicated by shading. The terminal Ala-Ala motif conserved among σ^{AntA} orthologues is bolded. Clustal Ω program available at: http://www.ebi.ac.uk/Tools/msa/clustalo/ was developed by Sievers *et al*., 2011.

Fig. S3. σ^{AntA} comprises a new ECF RNA polymerase σ factor subfamily. σ^{AntA} proteins were aligned to random proteins from each ECF RNA polymerase σ factor subfamily reported by (Staro *et al*., 2009) using ClustalΩ (Sievers et al., 2011) and PhyML (Guindon *et al.*, 2010) used to generate the phylogenetic tree, which was visualised using FigTree v1.4. All σ^{AntA} cluster together and possess a distinct lineage from all known ECF RNA polymerase σ factor subfamilies.

Fig. S4. Bacterial two-hybrid analysis did not detect an interaction between σ^{AntA} and the putative transmembrane protein STRS4_02195. β-galactosidase activity measured for DHM1 cells harbouring empty pKT25 plasmid (black bars), pKT25-*antA* (dark grey bars), pKT25-*STRS4_02195* (light grey bars), pKT25-*sigB* (white bar), pKT25-zip (hashed bar) and the indicated pUT18C plasmid are shown. Each bar represents the mean value of enzyme activities (represented as Miller units) from three technical replicates and the standard error is also shown. The plasmid pair pKT25-zip + pUT18C-zip is a positive procedural control.

Figure S1. The antimycin gene cluster is organised into four transcriptional units

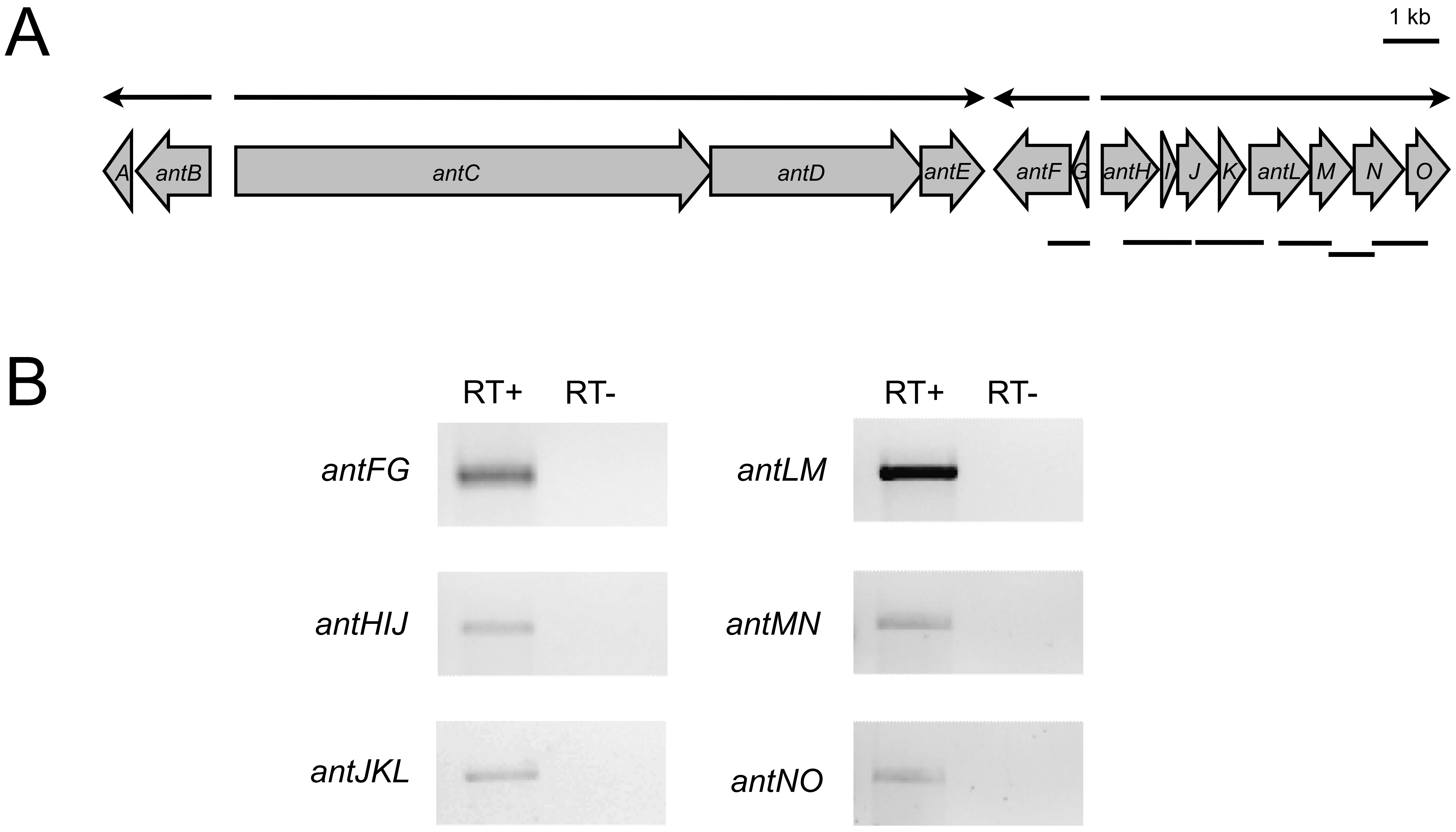

Figure S2. Clustal Ω alignment of AntA orthologues

```
S. blastmyceticus        VRTLNDPCAPPVTPLLRRAQAGDRQAMNDLLLHIAPYVGRLCLPIAQGHSSDAAQEAMLA
S. albus S4              MNTAHEL-PAPVDRLLPRARAGDEQAMNDLLLHITPYVTRVCRSVAHDNGSDARQEALLA
S. SM8                   MNTAHEL-PAPVDRLLPRARAGDEQAMNDLLLHITPYVTRVCRSVAHDNGSDARQEALLA
S. albus J1074           MNTAHEL-PAPVDRLLPRARAGDEQAMNDLLLHITPYVTRVCRSVAHDNGSDARQEALLA
S. NRRL 2288             MNTAHEL-PAPVDRLLPRARAGDEQAMNDLLLHITPYVTRVCRSVAHDNGSDARQEALLA
S. lappah-202            MNTAHEL-PAPVDRLLPRARAGDEQAMNDLLLHITPYVTRVCRSVAHDNGSDARQEALLA
S. CNY228                MNTAHEL-PAPVDRLLPRARAGDEQAMNDLLLHITPYVTRVCRSVAHDNGSDARQEALLA
S. gancidicus            MKTAHEP-TTLLPALLRQAQLGDEQAMNDLLTRITPYVARICLSIAHDNSSDATQEALLA
S. 303MF                 MSNVSDI-P-TPLALLPRAQAGDEQAMNQLLTGIVPYVARLCRSITHDDGADATQEALLA
S. hygroscopicus 5008    MSTVSEL-A-SPLTLLPRAQSGDEHAMNHLLNHITPYVNRICRSITRDDAADATQEALLA
S. hygroscopicus TL01    MSTVSEL-A-SPLTLLPRAQSGDEHAMNHLLNHITPYVNRICRSITRDDAADATQEALLA
S. griseoflavus          MTTVSEL-P-SALTLLPRAQAGDEHAMNHLLTAITPYVARICRSITHDDGADATQEALLA
S. TOR3209               MTTVSEL-P-SPLTLLPRAQAGDEHAMNHLLTGITPYVARICRSITHDDGADATQEALLA
S. ambofaciens           MSTVSEL-P-SPLMLLPRAQAGDEQAMNRLLTGITPYVARICRSITHDDGADATQEALLA
                         : .  :        ** :*: **.:*** **  *.*** *:*  ::: ..:** ***:**

S. blastmyceticus        IYRGIRGLREPAAFYGWVRAVTVREAIRTSKRLSQGSADGLPDLAQDADPLDTVHISDVM
S. albus S4              IYRGLPGLREPAAFYGWVRSVTVREAVRTARRLGRETTSPEVDSRHEPHSLDAVHINDVL
S. SM8                   IYRGLPGLREPAAFYGWVRSVTVREAVRTARRLGRETTSPEVDSRHEPHSLDAVHINDVL
S. albus J1074           IYRGLPGLREPAAFYGWVRSVTVREAVRTARRLGRETTSPEVDSRHEPHSLDAVHINDVL
S. NRRL 2288             IYRGLPGLREPAAFYGWVRSVTVREAVRTARRLGRETTSPEVDSRHEPHSLDAVHINDVL
S. lappah-202            IYRGLPGLREPAAFYGWVRSVTVREAVRTARRLGRETTSPEVDSRHEPHSLDAVHINDVL
S. CNY228                IYRGLPGLREPAAFYGWVRSVTVREAVRTARRLGRETTSPEVDPRHEPHSLDAVHINDVL
S. gancidicus            VYRGLAGLREPAAFYGWVRSVTVREAVRTAKRLGDTVPCSPVEARYEANPLDTVYINDVL
S. 303MF                 VYRGLRTLREPTAFYGWVRSVTVREAVRTVKRAGAEGPYQRVEPGQEANPLDAVHISDVL
S. hygroscopicus 5008    IYRGLGQLREPVAFYGWVRAVTVREALRTAKRFGEEQTCGQVESQQQANPLDAVHISDVL
S. hygroscopicus TL01    IYRGLGQLREPVAFYGWVRAVTVREALRTAKRFGEEQTCGQVESQQQANPLDAVHISDVL
S. griseoflavus          VYRGLGSLREPAAFYGWVRSVSVREAVRTAKRYGTERCCSEVEARQETNPMDAVYISDVL
S. TOR3209               IYRGLRTLREPAAFYGWVRSVTVREAVRTAKRYGEERSCCELETRQETNPLDAVHISDVL
S. ambofaciens           IYRGLGSLREPAAFYGWVRSVTVREAVRTAKRYGEERSCSELESRQEANPLDAVHISDVL
                         :***:  ****.*******:*:****:** :* .        :   : . :*:*:*.**:

S. blastmyceticus        DRLPDQHRQILTLRAVYGMNEQEMAATLSLPVGTVRSRLHRARRNFQDAWHQRAA
S. albus S4              DRMSEAHRQVLTLR-VYGLNEVEMAETLALPVGTVRSRLHRARRRFQEAWQPTAA
S. SM8                   DRMSEAHRQVLTLR-VYGLNEVEMAETLALPVGTVRSRLHRARRRFQEAWQPTAA
S. albus J1074           DRMSEAHRQVLTLR-VYGLNEVEMAETLALPVGTVRSRLHRARRRFQEAWQPTAA
S. NRRL 2288             DRMSEAHRQVLTLR-VYGLNEVEMAETLALPVGTVRSRLHRARRRFQEAWQPTAA
S. lappah-202            DRMSEAHRQVLTLR-VYGLNEVEMAETLALPVGTVRSRLHRARRRFQEAWQPTAA
S. CNY228                DRMSEAHRQVLTLR-VYGLNEVEMAETLALPVGTVRSRLHRARRRFQEAWQPTAA
S. gancidicus            ERLSTAHREVLTLR-VYGLNEEEMAETLSLPIGTVRSRLHRARRRFQEAWQPSAA
S. 303MF                 DRLSMSHRQVLTLR-AYGLNEEEMARVLSLPVGTVRSRLHRARLRFREAWQPSAA
S. hygroscopicus 5008    DRLSRAHRQVLTLR-AYGLNEEEIAEVLALPVGTVRSRLFRARRRFQEAWQPSAA
S. hygroscopicus TL01    DRLSRAHRQVLTLR-AYGLNEEEIAEVLALPVGTVRSRLFRARRRFQEAWQPSAA
S. griseoflavus          ERLSQAHRQVLALR-VYGLNEEEMAEVLALPVGTVRSRLFRARRRFQEAWQPSAA
S. TOR3209               ERLSQAHRQVLTLR-AYGLTEDEIAEVLALPLGTVRSRLFRARRRFQEAWQPSAA
S. ambofaciens           ERLSQAHRQVLTLR-AYGLNEEEMAEVLALPVGTVRSRLFRARRRFQEAWLPSAA
                         :*:   **::*:** .**:.* *:* .*:**:*******.*** .*::**   **
```

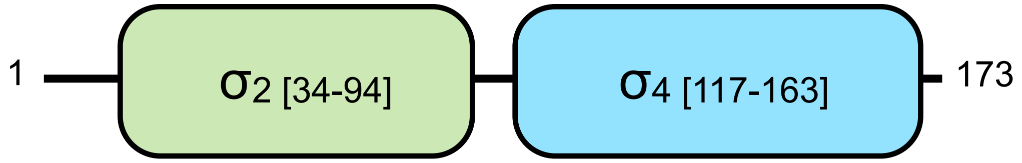

Figure S3. σ^{AntA} comprises a new subfamily of ECF RNA polymerase σ factors

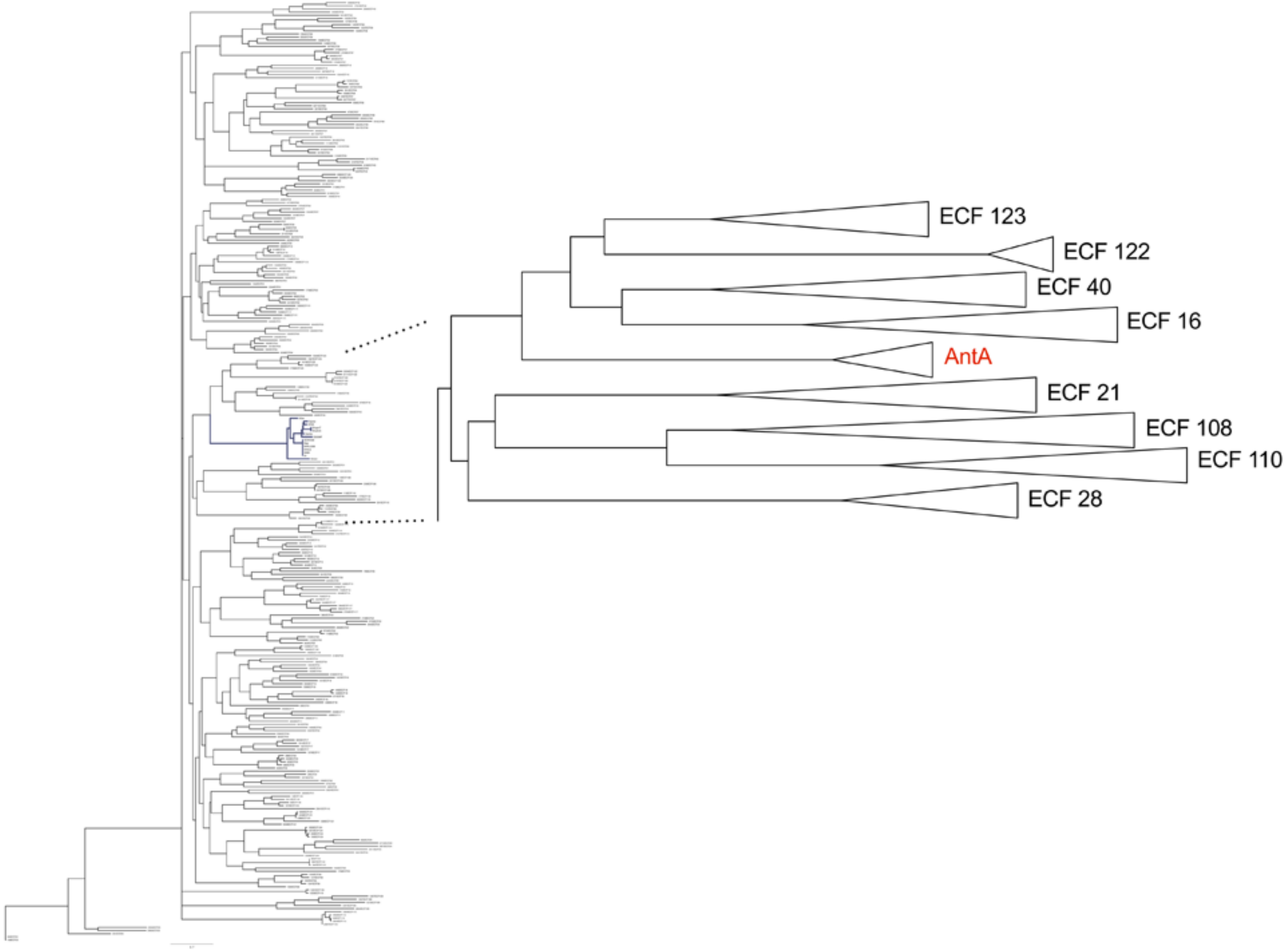

Figure S4. σ^{AntA} does not interact with STRS4_02195

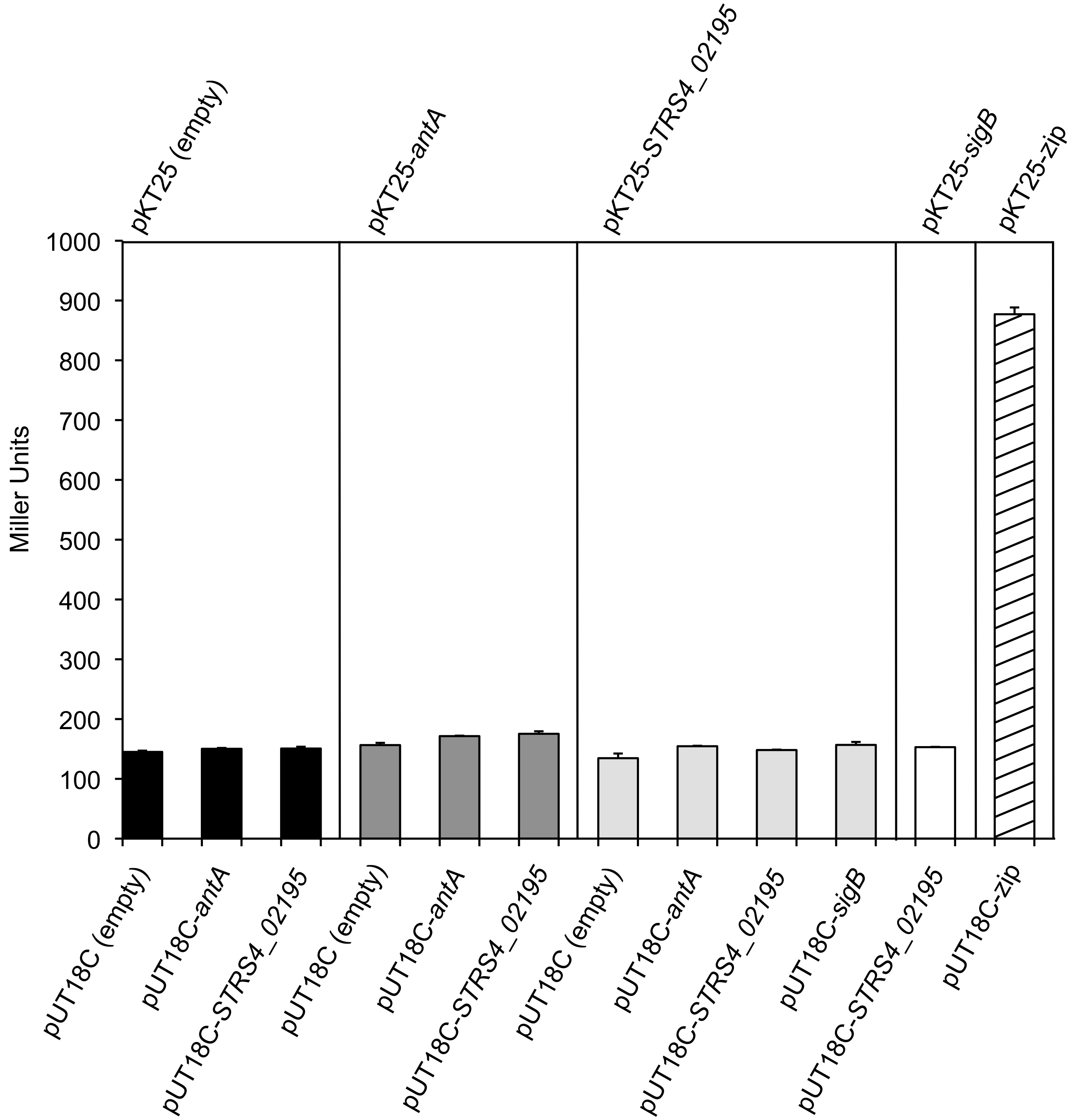

Table S1 tblastn analysis suggests that σ^{AntA} is only encoded by antimycin-producers

Species	Identity to σ^{AntA}	AA motif ?	Predicted targets	Source	E-value
Streptomyces albus J1074	100%	Yes	Antimycin biosynthesis	tblastn	3.00E-100
Streptomyces sp. SM8	100%	Yes	Antimycin biosynthesis	tblastn	3.00E-100
Streptomyces sp. NRRL 2288	100%	Yes	Antimycin biosynthesis	Yan et al 2012	3.00E-100
Streptomyces sp. LaPpaH-202	100%	Yes	Antimycin biosynthesis	tblastn	3.00E-100
Streptomyces sp. CNY228	99%	Yes	Antimycin biosynthesis	tblastn	2.00E-99
Streptomyces ambofaciens ATCC 23877	76%	Yes	Antimycin biosynthesis	Choulet et al 2006	1.00E-68
Streptomyces sp. 303MFCol5.2	69%	Yes	Antimycin biosynthesis	tblastn	8.00E-67
Streptomyces gancidicus BKS 13-15	74%	Yes	Antimycin biosynthesis	Kumar et al 2013	2.00E-66
Streptomyces sp. TOR3209	73%	Yes	Antimycin biosynthesis	Hu et al 2012	4.00E-66
Streptomyces griseoflavus Tu4000	73%	Yes	Antimycin biosynthesis	tblastn	3.00E-66
Streptomyces hygroscopicus subsp. jinggangensis TL01	72%	Yes	Antimycin biosynthesis	tblastn	9.00E-65
Streptomyces hygroscopicus subsp. jinggangensis 5008	72%	Yes	Antimycin biosynthesis	tblastn	9.00E-65
Streptomyces blastmyceticus NBRC 12747	66%	Yes	Antimycin biosynthesis	Yan et al 2012	1.00E-59

Table 2. Oligonucleotides used in this study

Primer alias	Sequence (5'-3')*	Description
RFS172	**aagctt** acgatctggtcctcgggctc	PCR: screening cosmid library for *antC*-containing cosmids
RFS173	**ggatcc** cagccggtggaaggtgac	PCR: screening cosmid library for *antC*-containing cosmids
RFS184	ccattattatcatgacattaa	End sequencing of Supercos1 cosmids
RFS185	gtccgtggaatgaacaatgg	End sequencing of Supercos1 cosmids
RFS186	cacgcgcccgcgtgtctcaccccgccatggtggcc<br>gtcatgtaggctggagctgcttc	PCR: *antA* REDIRECT knockout cassette
RFS187	cgcccgcaccgtcctccccgcaaggagagcaccc<br>ccatgattccggggatccgtcgacc	PCR: *antA* REDIRECT knockout cassette
RFS188	cttgcggggaggacggtgcgggcgggcggtccgc<br>ggtcatgtaggctggagctgctc	PCR: *antB* REDIRECT knockout cassette
RFS189	tgccaagatcggggcctttcgctgtctcccctcgccg<br>tgattccggggatccgtcgacc	PCR: *antB* REDIRECT knockout cassette
RFS222	gtacccccgccccctttcccccgattgggtacctcca<br>tgattccggggatccgtcgacc	PCR: *STRS4_0213-17* REDIRECT knockout cassette
RFS223	cacgtgtgcgcgggcaccctggccccgtccggcgt<br>ctcatgtaggctggagctgcttc	PCR: *STRS4_0213-17* REDIRECT knockout cassette
RFS226	ctccgtcgcgcacccacggatcagaaggggacac<br>acatgattccg gggatccgtcgacc	PCR: *STRS4_02194* REDIRECT knockout cassette
RFS227	gcgaggccggccgccgggaagacctgaggccc<br>gtgatcatgtaggctggagctgcttc	PCR: *STRS4_02194* REDIRECT knockout cassette
RFS228	acggctggaccgagatcgtcta	PCR: Δ*STRS4_02194* knockout confirmation
RFS229	tgttccgcgcaccgtcctca	PCR: Δ*STRS4_02194* knockout confirmation
RFS230	ata**catatg**aacaccgcgcacgaact	PCR: AntA coding sequence for complementation plasmid
RFS231	ata**aagctt**tcaggcggcggtgggctgcc	PCR: AntA coding sequence for complementation plasmid
RFS232	cccttgccgtcagagagatc	PCR: confirmation of Δ*antA* mutant strain
RFS233	gcttcaccccgcagtacatg	PCR: confirmation of Δ*antA* mutant strain
RFS258	acagcctgaccgccatggaa	PCR: confirmation of Δ*STRS4_0213-17* mutant strain
RFS259	actgaccgaggacgtcgtacgtgag	PCR: confirmation of Δ*STRS4_0213-17* mutant strain
RFS260	cctcgttcaggccgtaca	RT-PCR: *antA*
RFS261	acgaaccccactcactcg	RT-PCR: *antA*
RFS262	atcgtcacggtcaggaggt	RT-PCR: *antB*
RFS263	gcgtacatcgaggaactgaac	RT-PCR: *antB*
RFS264	gtcaacatcgtcccccttcag	RT-PCR: *antC*
RFS265	accgagatctggaggtcgt	RT-PCR: *antC*
RFS266	agtacatgtgccacccgaag	RT-PCR: *antE*
RFS267	gccgaagagctgacggta	RT-PCR: *antE*
RFS268	ggacctcgccgtagtcct	RT-PCR: *antF*
RFS269	aacgtctacccctcggagat	RT-PCR: *antF*
RFS270	ctcgaccaggaggaccag	RT-PCR: *antG*
RFS271	ctcaccggtaccaccgagta	RT-PCR: *antG*
RFS272	gctgtgccacacctcgta	RT-PCR: *antH*
RFS273	agagcgccttcatcaggtc	RT-PCR: *antH*
RFS274	cacccgcaccaggtctac	RT-PCR: *antO*
RFS275	cagatgcatccggtcgta	RT-PCR: *antO*
RFS276	tacctggcgtccgcggggagacggcatgatcggc<br>ccatgattccg gggatccgtcgacc	PCR: *STRS4_02195* REDIRECT knockout cassette
RFS277	tttccggtgcccgcctcccgctcctgccgcgagaac<br>tcatgtaggctggagctgcttc	PCR: *STRS4_02195* REDIRECT knockout cassette
RFS278	ggtacgcgccctcccgac	PCR: confirmation of Δ*STRS4_02195* mutant strain
RFS279	ctttccggtgcccgcctc	PCR: confirmation of Δ*STRS4_02195* mutant strain

RFS280	ata**ggatcc**aatgcccagggaacgcgtact	PCR: STRS4_02195 coding sequence for bacterial two-hybrid plasmids
RFS281	ata**ggtacc**ttcgcggctccgccggagccgg	PCR: STRS4_02195 coding sequence for bacterial two-hybrid plasmids
RFS282	ata**ggatcc**aatgaacaccgcgcacgaac	PCR: AntA coding sequence for bacterial two-hybrid plasmids
RFS283	ata**ggtacc**ttggcggcggtgggctgccatg	PCR: AntA coding sequence for bacterial two-hybrid plasmids
RFS284	ata**ggatcc**aatgtccgcacagactgaagc	PCR: STRS4_04339 (SigB orthologue) coding sequence for bacterial two-hybrid plasmids
RFS285	ata**ggtacc**ttctcctcggcgagcatccccg	PCR: STRS4_04339 (SigB orthologue) coding sequence for bacterial two-hybrid plasmids
RFS286	cgtcgctgggcgcagtggaacgcc	PCR: sequencing pUT18C inserts
RFS287	cttaactatgcggcatcagagc	PCR: sequencing pUT18C inserts
RFS288	gttcgccattatgccgcatc	PCR: sequencing pKT25 inserts
RFS289	ggatgtgctgcaaggcgatt	PCR: sequencing pKT25 inserts
RFS298	gtcgacggtgacgcccttgg	RT-PCR: *antFG* co-transcription
RFS299	atgagcaccatcagggagttgct	RT-PCR: *antFG* co-transcription
RFS302	tcaccaacgcgctgctcgtc	RT-PCR: *antJKL* co-transcription
RFS303	ggccgtccggaacctccag	RT-PCR: *antJKL* co-transcription
RFS304	gctgaaggacgcgtacgtcg	RT-PCR: *antLM* co-transcription
RFS306	gctgtcggggacgggttcc	RT-PCR: *antMN* co-transcription
RFS307	ggagtcgttgagcgcggaga	RT-PCR: *antMN* co-transcription
RFS308	gcgtgaggcgttggtcaagg	RT-PCR: *antNO* co-transcription
RFS309	acgtcgtcgaaggtctcggg	RT-PCR: *antNO* co-transcription
RFS329	gctctggattctcaacaatgc	PCR: confirmation of Δ*STRS4_02212* mutant strain
RFS330	tgaggcggcgattgatt	PCR: confirmation of Δ*STRS4_02212* mutant strain
RFS317	agcgcgttcatgaaggtgat	RT-PCR: *antAB* operon 5'RLM-RACE
RFS318	cccgccctccctgcggatca	RT-PCR: *antAB* operon 5'RLM-RACE
RFS323	agctctccaccacctcgat	RT-PCR: *antGF* operon 5'RLM-RACE
RFS324	caactgggtcagccacatca	RT-PCR: *antGF* operon 5'RLM-RACE
RFS340	tcaagcggcacaccaacgac	RT-PCR *antHIJ* co-transcription
RFS341	atggtcggcgccctggtgat	RT-PCR *antHIJ* co-transcription
RFS342	tggtcgtggccgtagtgga	RT-PCR: *antLM* co-transcription
RFS345	cgccgagtacaggtaca	RT-PCR: *antHIJKLMNO* operon 5'RLM-RACE
RFS346	ctgcatcccgatgatct	RT-PCR: *antHIJKLMNO* operon 5'RLM-RACE
RFS351	ata**gaattc**ggcgggagaaaaga	PCR: *antA* native promoter complementation plasmids
RFS352	ata **ggatcc**tcagtcgtcggtgggctgc	PCR: *antA* native promoter complementation plasmid and introduces A172D A173D point mutations

* non-homologous sequences are underlined and engineered restriction endonuclease sites are bolded
